# Python farming as a flexible and efficient form of agricultural food security

**DOI:** 10.1038/s41598-024-54874-4

**Published:** 2024-03-14

**Authors:** D. Natusch, P. W. Aust, C. Caraguel, P. L. Taggart, V. T. Ngo, G. J. Alexander, R. Shine, T. Coulson

**Affiliations:** 1 School of Natural Sciences, Macquarie University, Sydney, NSW 2109 Australia; 2 Department of Zoology, University of Oxford, Oxford, UK; 3 Animal, Plant and Environmental Sciences, University of the Witwatersrand, Johannesburg, South Africa; 4 School of Animal & Veterinary Science, The University of Adelaide, Adelaide, SA 5371 Australia; 5 National Key Laboratory, Institute of Tropical Biology, Vietnamese Academy of Sciences and Technology, 9/621 Hanoi Highway, Thu Duc City, Ho Chi Minh City, Vietnam

**Keywords:** *Malayopython reticulatus*, *Python bivittatus*, Reptile, Snake, Sustainable food production, Climate change resilience, Animal physiology, Herpetology

## Abstract

Diminishing natural resources and increasing climatic volatility are impacting agri-food systems, prompting the need for sustainable and resilient alternatives. Python farming is well established in Asia but has received little attention from mainstream agricultural scientists. We measured growth rates in two species of large pythons (*Malayopython reticulatus* and *Python bivittatus*) in farms in Thailand and Vietnam and conducted feeding experiments to examine production efficiencies. Pythons grew rapidly over a 12-month period, and females grew faster than males. Food intake and growth rates early in life were strong predictors of total lifetime growth, with daily mass increments ranging from 0.24 to 19.7 g/day for *M. reticulatus* and 0.24 to 42.6 g/day for *P. bivittatus*, depending on food intake. Pythons that fasted for up to 4.2 months lost an average of 0.004% of their body mass per day, and resumed rapid growth as soon as feeding recommenced. Mean food conversion rate for dressed carcasses was 4.1%, with useable products (dressed carcass, skin, fat, gall bladder) comprising 82% of the mass of live animals. In terms of food and protein conversion ratios, pythons outperform all mainstream agricultural species studied to date. The ability of fasting pythons to regulate metabolic processes and maintain body condition enhances food security in volatile environments, suggesting that python farming may offer a flexible and efficient response to global food insecurity.

## Introduction

The raising of livestock is a cornerstone of human civilization, has underpinned the rise of global economies, and continues to play a central role in the well-being of people in many cultures^1–3^. Livestock production traditionally has relied on a small number of domesticated species and production models—a little-changed formula that until now has served humanity well^2^. A central characteristic of conventional livestock systems has been a high rate of production, driven by energy intensive endothermic (warm-blooded) animals^2,4^. High performance endothermic physiologies generating nutrient-dense food, and cheap horsepower in the form of draft animals were important enablers for early civilisations^2^. Essential feed inputs were sustained by primary productivity, and livestock systems often developed within a context of resource abundance and stability^5,6^. These parameters are now no longer the norm, and the twin challenges of resource limitations and climate volatility are rapidly changing the production imperatives of our food systems^7–9^.

Conventional livestock and plant crop systems are faltering. Twelve percent of the global human population is undernourished and acute protein deficiency in low-income countries is compromising workforce productivity and development^10–12^. Global food security is predicted to worsen with global change^10^. Infectious diseases, diminishing natural resources, and climate change are having significant and compounding impacts on the agricultural sector^13–15^. Many conventional livestock systems fail to satisfy the criteria for sustainability and/or resilience, and there is an urgent need to explore alternatives^13^.

Ectotherms (cold-blooded animals) are approximately 90% more energy efficient than endotherms^16^. In the context of agriculture, this energy differential readily translates into a potential for higher production efficiency^17,18^. It is partly for this reason that the aquaculture and insect farming industries are currently experiencing rapid growth rates^17,19^. Like insects, snakes are a traditional source of protein in many tropical countries^20,21^, and their consumption is linked to important food, medicinal, and cultural values^22–24^. As demand for snake meat and co-products has increased in line with development, so too have production systems. Over the last two decades, snake farming has expanded to include more species, production models, and markets, partly as a result of competitive agricultural advantages^20^. For example, some snake production systems require minimal land and freshwater, they can rely on waste protein from other industries, and some snake species have specialised adaptations for mitigating the impacts of environmental shocks^20,25–27^. Another reason for recent expansion is appeal. Reptile meat is not unlike chicken: high in protein, low in saturated fats, and with widespread aesthetic and culinary appeal^22,28–30^.

We examined the potential of pythons as a novel form of livestock for commercial agriculture. To achieve this aim, we studied the growth patterns of two python species in two commercial farms in Southeast Asia. We assessed growth rates of juvenile snakes and conducted feeding experiments on a subset of the snakes to assess production efficiencies and key variables influencing growth. We compared the data gathered during our study to the results of research on other agricultural species (both endothermic and ectothermic) to assess the potential of commercial pythonfarming to enhance food security in the context of global change.

## Materials and methods

We collected data from two Asian python farms within the natural range of the model species used in this study: one in Uttaradit Province, central Thailand (17° 38′ N, 100° 07′ E) and the other in Ho Chi Minh City, in southern Vietnam (10°58'N, 106°30'E). The farm in Thailand farms both reticulated (*Malayopython reticulatus*) and Burmese (*Python bivittatus*) pythons, whereas the Vietnamese farm produces only the latter. Both Burmese and reticulated pythons are large-bodied (can grow to > 100 kg), fast growing , and highly fecund, with females reaching maturity within 3 years and producing up to 100 eggs per year for 20 years or more^31^. They are thus well suited for commercial production.

In both Thailand and Vietnam, pythons are housed in enclosures situated within warehouses. The warehouses are constructed and managed in a semi-open fashion to facilitate ventilation and provide optimal temperatures. We did not record the temperature of pythons or their enclosures during this study, but temperatures anecdotally varied between 25 and 32 °C. Pythons were housed communally at stocking densities of approximately 15 kg per m^2^. Captive-bred pythons in Thailand and Vietnam were fed on a variety of food types depending on local protein resources. The most common feed inputs were wild-caught rodents and waste protein from agri-food supply chains (e.g., pork, chicken, fish^20,32^). Many of the larger python farms make sausages from processed waste protein. Sausages are typically introduced into the diet only after the young pythons have developed a robust feeding response^32^.

### Trials of growth rate

To quantify growth rates and related attributes, individual pythons were repeatedly measured over a 12-month period. Most pythons are grown for 1 to 1.5 years before slaughter for meat, skins, and other products^32^. At each farm we collected hatchling pythons from eggs produced and hatched onsite. To identify individual snakes, we either maintained a photographic database of the skin patterns on a dorsolateral section of skin immediately posterior to the head, or implanted snakes with passive integrated transponder (PIT) tags^33^.

In Thailand, we measured snout-vent length (hereafter, SVL; using a flexible measuring tape run along the spine of each snake) and body mass (to the nearest g) of pythons on three occasions over the 12-month growing period: (1) at hatching, (2) at six months of age, and (3) at 12 months of age. We sexed snakes by inserting a lubricated probe into the cloacal bursae and recording depth. Pythons were each offered a frozen-thawed day-old chicken to eat once per week for the first two months. From two to 12 months pythons were offered a combination of frozen-thawed day-old chickens and sausages on a weekly basis (Table 1). The mass of food offered was not measured but was estimated to be less than 15% of the snakes’ bodyweight per feeding event.

**Table 1:** Details of site, purpose of study, and basic husbandry details of Burmese (*Python bivittatus*) and reticulated (*Malayopython reticulatus*) pythons in this study.

Site	Species	N	Traits measured	Food type	Feeding regime
Thailand	*P. bivittatus*	2381	Growth rate	Chickens and sausages	One feed offering per week
	*M. reticulatus*	2004	Growth rate	Chickens and sausages	One feed offering per week
Vietnam	*P. bivittatus*	60	Food conversion rate and fasting	Experimental (varied)	One feed offering every five days
	*P. bivittatus*	156	Growth rate	Rodents and chickens	One feed offering every five days

In Vietnam, we measured SVL and body mass of pythons at six intervals over a 12-month period (approximately 0, 2, 4, 7, 9, and 12 months of age) and sexed snakes at hatching by eversion of the hemipenes. Non-hatchling pythons were fed pork-based sausages or experimental diets (see below; Table 1). Feeding regimes followed farm protocols for maximum growth rates (food equal to ~ 15% of body mass provided once every 5 days). Hatchlings were started on vertebrate prey (i.e., rodents, day-old quail, or day-old chickens). Apart from one of the experimental groups, the diet of all snakes in treatment groups was changed to sausages at approximately two months of age.

### Intensive trials on growth rate

To quantify the influence of food intake on growth rates of pythons and to better quantify food-conversion efficiencies, we conducted a detailed feeding trial on a subset of Burmese pythons at the Vietnamese farm. When python eggs hatched, we divided snakes into five experimental groups, each comprising seven males and seven females (14 snakes total per treatment); we used systematic random allocation to distribute individuals among treatment groups (to deconfound treatments from maternal effects). Diet treatments included: (1) 100% pork; (2) 90% pork, 10% chicken pellets; (3) 90% pork, 10% fish pellets; (4) 80% pork, 20% fish pellets; and (5) 100% wild-caught rodents. We chose these diet treatments because they reflected those currently used in the python farming industry^20,32^. Rodents were sourced from local rice fields via professional trappers^20^ and humanely euthanized immediately prior to being fed to the pythons. The pork used in the sausages comprised of still-born piglets obtained from local farms, defrosted in vats of water then ground in an industrial meat grinder. The dry pellets used were commercial catfish and chicken grower pellets (31% and 16% protein, respectively) made predominantly from processed anchovies and rice-bran (Ha Lan Aquafeed, Viet Nam). The dry pellets were added to the pork immediately after grinding to facilitate rehydration. The homogenised paste was reconstituted into appropriately sized sausages using a commercial sausage-making machine.

Pythons were offered food approximately once every five days throughout the year, except for three months over the coldest period of the year when they were offered food less often. At each feeding event, we weighed each sausage before one or more was offered to the pythons. Food was offered using forceps and snakes were never force-fed. We recorded whenever snakes refused food. After feeding, pythons were weighed to calculate the pre-feeding body mass of each snake. This procedure allowed us to not disturb the pythons before feeding, ensuring a natural feeding response. We did not measure SVL due to the potential impact of handling stress on feeding behaviour. All study animals were provided drinking water ad libitum*.*

### Fasting

During the growth trials in Vietnam, we recorded the length of time pythons went without food to examine the influence of fasting on growth and mass loss. Depending on prey size, pythons typically digest meals within two weeks (range = 4–13 days^34^). To eliminate the influence of energy derived from previous food consumption we only considered pythons to be fasting if they had gone without food for at least 20 consecutive days. We used body mass measurements derived from feeding records before *versus* after fasting to calculate total mass lost during the fasting event. We divided total mass lost during the fasting event by the duration of each event to calculate mean mass loss per day.

### Carcass processing

At the completion of the intensive growth trials, pythons were humanely killed using standard procedures (i.e., captive bolt pistol^35^) and processed to record carcass characteristics. We dissected and weighed parts of the snake that are of commercial value, including the fat, gall bladder, skin, and dressed carcass (excluding head, tail, visceral organs and skin). We weighed the remaining organs and tissues to calculate the percentage of each item relative to total body mass. Finally, we calculated food conversion ratios (FCR) by dividing the total amount of food consumed by the mass of the dressed carcass.

### Analysis of data

**Figure 1:**
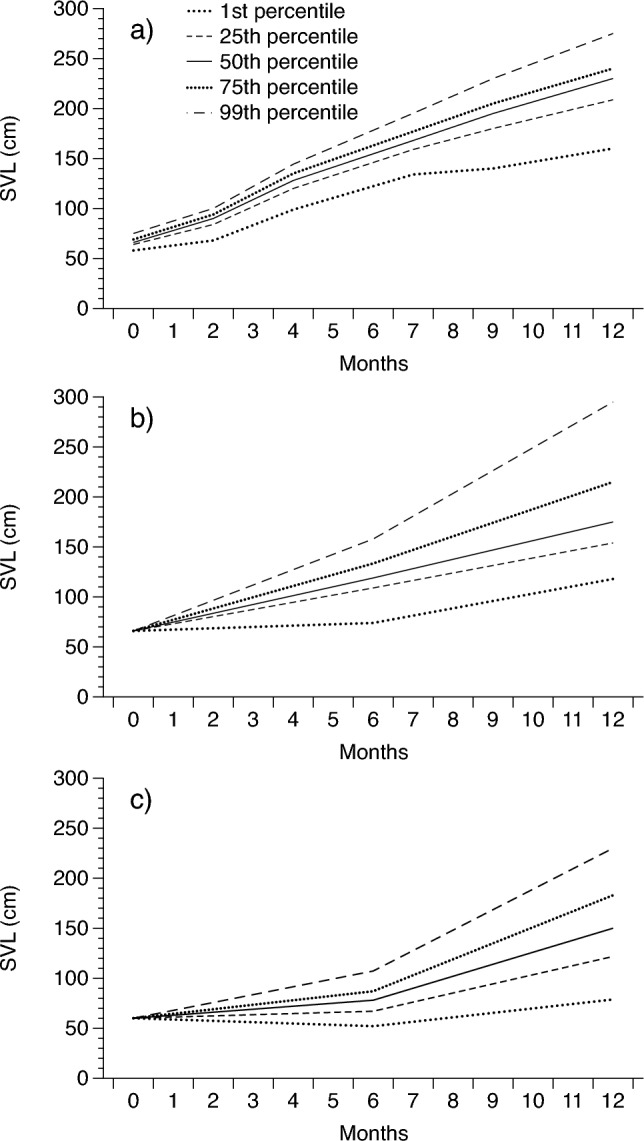
Change in snout-vent length (SVL) over time of (**a**) Burmese pythons in Vietnam, (**b**) reticulated pythons in Thailand, and (**c**) Burmese pythons in Thailand over a 12-month period. Solid lines show calculated averages (50th percentile) whereas dotted lines show other percentile values.

Because commercially valuable products from pythons are sold by mass, we mostly report rates of growth in body mass; however, in Fig. 1 and Table 2 we also provide growth rates as changes in SVL. Body mass in snakes is strongly correlated with SVL^36^. To assist with visualisation, we plotted variation in growth rates of individual pythons by presenting the growth rates of the 1^st^, 25^th^, 50^th^, 75^th^, and 99^th^ growth percentiles for pythons in each treatment. However, for our analysis we used the mean rate of growth from hatching to slaughter. We examined the influence of farm site and sex on growth rates for each python species in our non-intensive growth trials (separately) using a two-way analysis of variance (ANOVA) with sex and site (and their interaction) as factors, and growth rate over 12 months as the dependent variable.

**Table 2:** Mean ± standard errors and ranges (in parentheses) for daily growth rates of snout-vent length (SVL) and body mass for male and female reticulated (*Malayopython reticulatus*) and Burmese (*Python bivittatus*) pythons raised in farms in Thailand and Vietnam.

	N	Male		Female		Sexes combined	
		SVL (cm/day)	Mass (g/day)	SVL (cm/day)	Mass (g/day)	SVL (cm/day)	Mass (g/day)
Thailand							
* M. reticulatus*	2004	0.47 ± 0.002 (0.23–0.64)	4.1 ± 0.07 (0.24–19.1)	0.5 ± 0.003 (0.29–0.71)	6.3 ± 0.15 (0.28–19.7)	0.48 ± 0.002 (0.23–0.71)	5.2 ± 0.09 (0.24–19.7)
* P. bivittatus*	2381	0.37 ± 0.002 (0.03–0.68)	2.9 ± 0.06 (0.24–8.4)	0.42 ± 0.003 (0.03–0.84)	3.9 ± 0.08 (0.26–14.2)	0.4 ± 0.002 (0.03–0.8)	3.5 ± 0.05 (0.24–14.2)
Vietnam							
* P. bivittatus*	156	0.59 ± 0.008 (0.37–0.72)	18 ± 0.78 (3.2–37.2)	0.6 ± 0.008 (0.43–0.74)	19.9 ± 0.99 (3.7–42.6)	0.6 ± 0.006 (0.37–0.74)	19 ± 0.64 (3.2–42.6)
* P. bivittatus*	60	NA	10.2 ± 0.49 (4.0 –16.3)	NA	10.4 ± 0.61 (5.4–17.3)	NA	10.3 ± 0.38 (4.0–17.3)

No data on SVL are available for one of the Vietnamese treatments. See text and Table 1 for details of husbandry procedures for each species at each location.

To explore the factors influencing variability in growth rates of Burmese pythons in our intensive trial, we modelled mean 12-month growth rate against five attributes of the pythons and their husbandry (ln mass at birth, diet, ln 2-month growth rate, total amount of food consumed, and days spent fasting) in a multiple regression. We used a model selection approach to rank all possible models (and two-way interaction terms) based on AIC_c_ values^37^. We applied a one-way ANOVA with food type as the factor and growth rate over 12 months as the dependent variable to explore the potential influence of food type on rate of growth.

In some cases, our fasting dataset contained pythons that underwent multiple fasting events. We tested for differences in the likelihood of fasting between diet treatments using contingency table analysis. To account for pseudoreplication and the influence of individual-specific growth rates in our analysis, we analysed the influence of fasting duration, and the influence of mass prior to fasting, on the rate of loss of body mass using a generalized linear mixed model incorporating individual python ID as a random effect. We ln-transformed our data wherever necessary to meet the normality and homogeneity of variance assumptions required for our statistical tests, and conducted all analyses in JMP Pro 14 (SAS Institute: Cary, NC).

### Ethics statement

We stress that no snakes were harmed for the purpose of our study; we utilised existing farm operations and trade. Our data were gathered from snakes bred for a commercial industry, which employs humane methods of killing reptiles (by brain destruction^38^). All work was carried out with relevant permissions from the farm owners and authorities (Administration of Forestry of the Socialist Republic of Viet Nam 114/TCLN-CTVN). All procedures were approved by the Animal Ethics Screening Committee of the University of Witwatersrand, South Africa (approval number: 2014/17/B), the University of Adelaide, Australia (approval number: S-2018-084), were consistent with ARRIVE guidelines^39^, and all methods were performed in accordance with the relevant guidelines and regulations.

## Results

### Growth rates

**Figure 2:**
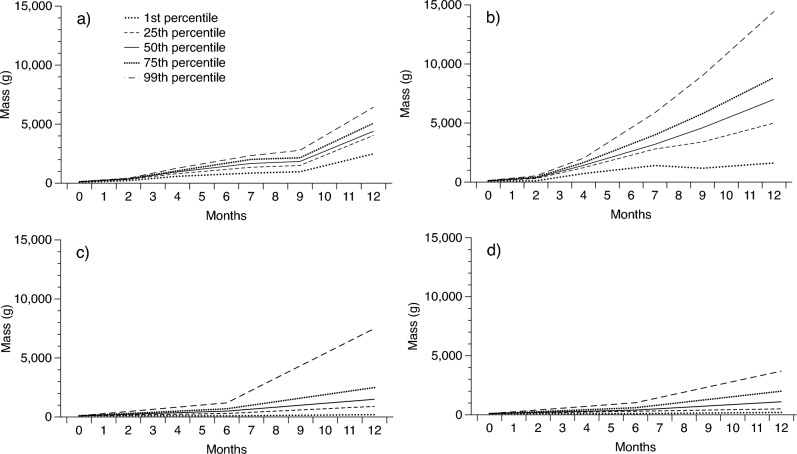
Change in body mass over time of (**a**) Burmese pythons in Vietnam in intensive trials, (**b**) Burmese pythons in Vietnam in rapid growth trials, (**c**) reticulated pythons in Thailand, and (**d**) Burmese pythons in Thailand. All data were gathered over a 12-month period. Solid lines show calculated averages (50th percentile) whereas dotted lines show other percentile values.

Both species of python grew rapidly at both farms (up to a maximum of 46 g/day; Tables 2, S1; Figs. 1, 2). Our ANOVA revealed that growth rates of Burmese pythons were slower at the Thai farm than at the Vietnamese farm (F_1,2591_ = 1005, *P* < 0.0001; Table 2) and females grew more rapidly than males at both farms (F_1,2591_ = 8.97, *P* < 0.0028; Table 2). Sex differences in growth did not vary between farms (interaction sex*site: F_1,2591_ = 2.01, *P* = 0.1559). At the Thai farm where both species were raised and husbandry and feeding procedures were similar, reticulated pythons grew faster than Burmese pythons held in the same facility (F_1,4526_ = 124, *P* < 0.0001) but still slower than Burmese pythons in Vietnam (Table 2; Fig. 2).

**Figure 3:**
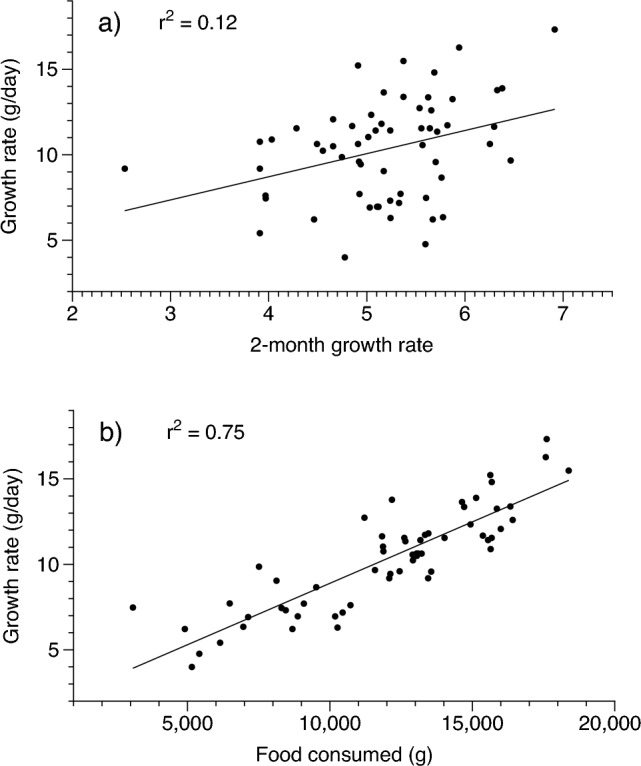
Relationship between growth rate over the first 12 months of life in captive Burmese pythons, and two significant predictors of that long term rate of growth: (**a**) growth rate over the first two months of life, and (**b**) total amount of food consumed over 12 months. See text for results of statistical significance tests.

Our most parsimonious model included growth rates over the first two months of life and the amount of food consumed (Fig. 3). Pythons that grew fastest in their first two months of life, and which consumed the most food, grew the fastest over the 12-month period (Figs. 1, 2, 3). The model with the best support that included only a single predictive variable included the amount of food consumed, confirming that food intake is the primary determinant of python growth rates (Fig. 3). A follow-up ANOVA with food type as the factor and ln 12-month growth rate as the dependent variable confirmed that the different food types provided as part of our experimental trials (Table S1) did not significantly influence growth rates in Burmese pythons (although this difference was close to statistical significance: F_4,54_ = 2.50, *P* = 0.054).

### Influence of fasting

Over the course of our intensive growth study, 61% (43/70) of Burmese pythons fasted for periods of 20 days or more (up to 127 days). Some pythons fasted multiple times throughout the study, for a maximum fasting duration of 228 days. Fasting was recorded in pythons from all diet regimes and although the proportion of pythons fasting was higher in animals fed on the two diets containing fishmeal, contingency-table analysis confirmed that the difference was not statistically significant (χ^2^_4_ = 5.7, *P* = 0.23). Mean daily mass loss during episodes of fasting was 0.16 ± 0.7 g per day. When calculated as a percentage of body mass prior to the fasting event, pythons lost an average of 0.004 ± 0.03% of their body mass per day. Some snakes gained body mass while fasting (likely due to drinking), and our mixed effects model showed no significant correlation between the rate of mass loss (or gain) and the duration of time spent fasting (F_1,72_ = 1.89, *P* = 0.174). Our mixed effects model also confirmed that larger pythons fasted for longer durations than did smaller conspecifics (F_1,72_ = 9.08, *P* = 0.0037). Although fasting did not result in a significant loss of body mass, it did reduce the total amount of food consumed, which significantly reduced overall growth rates.

### Food conversion ratios and useable products

Mean food conversion ratio for the 58 snakes followed throughout their lives was 4.1: 1 (4.1 ± 0.06 g; range 3.15–4.85). That is, pythons consumed an average of 4.1 g of food for every 1 g of dressed carcass produced. The mass of commercially valuable body parts obtained from each snake increased with the mass of the animal (and hence, with its growth rate; all correlations have *P* < 0.0001). After removal of non-useable organs, useable parts of the snake (including dressed carcass, gall bladder, fat, and skin) averaged 82 ± 0.8% (range: 69–90%) of overall snake mass (Table 3).

**Table 3:** Commercially useable and non-useable products from Burmese pythons (*Python bivittatus*) as a percentage of total mass of the carcass, based on a sample of 58 captive-raised pythons with a mean body mass of 5.6 kg (data for males and females combined).

Organ	Mean % (SE) of overall body mass (%)	Range (%)
Dressed carcass	54 ± 0.5	45–69
Gall bladder	0.3 ± 0.01	0.09–0.6
Fat	6.4 ± 0.2	1.7–9.8
Skin	22 ± 0.3	18–33
Non-useable organs	18 ± 0.8	10–31

## Discussion

An extensive literature documents fast growth rates for pythons, and our experimental trials confirm that pythons can grow very rapidly over their first year of life. Despite this ability, pythons have been overlooked as a mainstream agricultural species^40^. Instead, concerns have been raised that commercial production of these snakes in captivity is not feasible and that Asian farms are simply laundering wild-caught snakes under the guise of being captive-bred^41,42^. We have no data to support or refute the latter claim, but our studies confirm earlier work that it is biologically and economically feasible to breed and raise pythons in captive production facilities for commercial trade^32^. We first discuss the significance and limitations of our results before turning our attention to the assessment of pythons as a novel livestock species for commercial agriculture.

Growth rates in both python species that we assessed were highly plastic and were strongly influenced by the amount of food consumed. Although fasting resulted in slower growth, variation in growth rates was best explained by overall food intake rates; that is, fasting pythons grow slower due to reduced food intake, but there did not appear to be any additional growth cost to fasting per se. In keeping with other studies on snakes, body mass at hatching did not influence growth rates. Instead, a snake’s growth trajectory over the first two months of its life predicted its subsequent growth rate and hence its body size later in life^43^.

Females of both species grew faster than males. Although female-biased growth rates are common in snakes, growth rates in pythons do not typically diverge until after reaching maturity^44,45^. We detected sexual divergence in growth rates well before maturation, suggesting that sex-based divergences in growth rate divergence are subtle and may only be detectable with large sample sizes such as those used in our study.

Pythons grew faster in the Vietnamese farm than in the Thai farm, likely due to a more frequent feeding regime. Although reticulated pythons grew faster than Burmese pythons in the Thai facility, where both species are maintained, we are reluctant to conclude that this species exhibits faster overall growth rates in the wild, or that the growth potential of the reticulated python exceeds that of the Burmese python. Growth rates are very flexible and driven primarily by food consumption. Burmese pythons in both trials in Vietnam had faster growth rates than did Thai reticulated pythons, and overall snakes in Thailand were offered less food than their Vietnamese conspecifics.

Why were the costs of fasting (in terms of mass loss) so low? Pythons have specialised physiological and morphological responses to both feeding and fasting^46–48^. The gastrointestinal tract is adapted for long periods of quiescence punctuated by rapid metabolic upregulation for digestion and assimilation of large meals (sometimes, > 100% of body mass^49^). During digestion, pythons exhibit a tenfold increase in metabolic rate above resting levels; organ performance increases up to 40-fold; and circulating hormones and metabolites increase by as much as a 100-fold^46–48^. After digestion is completed, the process is reversed and metabolic functions are rapidly downregulated. Ingested macronutrients are partitioned and selectively oxidised in preparation for fasting^50^. Lipids are stored in specialised fat bodies and leveraged during fasting to fuel atrophic energy requirements^50,51^. Our study provides further evidence for these remarkable physiological processes and identifies their utilitarian potential in an agricultural context.

We now turn our attention to the agricultural potential of pythons as it relates to the biology of these snakes. As large-bodied, fast-growing ectotherms with flexible digestive physiologies, our study confirms that pythons have considerable agricultural potential. The pythons in our study were capable of high food conversion ratios and rapid growth rates, and can tolerate long periods of fasting without substantial loss of mass. The dietary treatments that we offered did not significantly influence growth rates of the snakes, suggesting that pythons exhibit efficient protein conversion ratios under a range of dietary and production scenarios. Our findings support previous studies highlighting the role of snake farms in facilitating efforts to control rodent pests, and in upcycling waste-protein resources to close nutrient cycle loops^20,21,32,52^.

Pythons are obligate carnivores, and thus belong to a trophic level (predators) that classical Lindeman trophic pyramids would regard as poorly suited to farming: that is, inefficient and environmentally unsustainable^53,54^. Our results suggest otherwise. Table 4 provides a comparison of some key production criteria in livestock systems. Production efficiencies for pythons were higher than those reported for poultry, pork, beef, salmon, and crickets (Table 4). This remarkable outcome reflects the synergistic effects of ectothermic physiology^16^, sessile behaviour^55^, efficient digestive physiology^56^, and economic serpentine morphology (e.g., no legs or wings ~ higher edible carcass ratio). High assimilation efficiencies also translate into low volumes of faeces, and the nitrogenous wastes that pythons produce are excreted as water-insoluble urates rather than more volatile urea^57^. Python farms, therefore, produce fewer greenhouse gasses (CO_2_, methane and nitrous oxide) than do endothermic livestock systems^58,59^.

**Table 4:** Comparisons of some key production metrics in livestock production systems.

Variable	Endotherms			Ectotherms		
	Poultry^60,61^	Pork^62–64^	Beef^65,66^	Salmon^67,68^	Cricket^18,69^	Python _*_^70^
Feed conversion ratio (DM feed : dressed carcass)	2.8	6.0	10.0	1.5	2.1	1.2
Protein DM in feed (%)	22	16	12	50	22	51
Protein conversion ratio	20.5	37.5	83.3	3.0	9.5	2.4
Fasting yield loss per hour (%; with access to water)	0.22	0.21	0.15	0.0055	No data	0.0002

“Feed conversion ratio” = total dry mass in food consumed by the animal divided by total dry mass of the carcass (so lower value represents higher efficiency of conversion). “Protein DM in feed” = dry mass of protein as a proportion of total dry mass of food. “Protein conversion ratio” = total dry mass of protein consumed in food divided by total dry mass of protein in carcass (so lower value represents higher efficiency of conversion). “Fasting yield loss per hour” = mass loss per hour over period when animal has access to water but no food.

*Calculated from this study. Based on whole pig waste protein diet, wet weight to dry mass conversion rate of 28.9^70^.

One caveat to the rapid growth rates reported here is that in one of our diet treatments (Burmese pythons in Vietnam), a significant proportion (~ 20%) of pythons died due to respiratory infections. Similar growth rates of pythons from a different treatment at the same farm, and from Thailand, did not result in such high mortalities (< 5%). It is not known what caused such a high incidence of respiratory infection in one year, but the experimental diet (e.g., possible micronutrient deficiencies) coupled with unseasonably cool weather may be contributing factors.

The ability of pythons to fast for extended periods without jeopardizing survival or body condition is remarkable. For example, five 6-month-old pythons ceased feeding for four months (approximately 45% of their lives) but only lost 30 to 70 g (2.7–5.4% of their pre-fasting body mass) over that period. Few other animals can downregulate metabolic costs to this degree, and species utilized by the mainstream agricultural industry certainly cannot do so (Table 4). That ability of pythons to maintain near-stasis in body mass over prolonged periods of food deprivation confers great flexibility for producers. Food systems resilience is closely linked to disruptions in supply chains and famine tolerance^15,71^. Pandemics and extreme weather events coupled with the inability of livestock to retain body condition in the absence of reliable feed supplies present increasing risks to food security. Pythons offer farmers the flexibility needed to regulate both feed inputs and product outputs in response to unpredictable external factors.

In addition to flexibility in feeding regimes and rapid growth rates, the natural history of at least some species of pythons is characterised by early maturity and high reproductive output^31,72^. Most species are ecological generalists, exploiting both above- and below-ground habitat niches to evade extreme weather events^27,55,73,74^. They can survive without fresh water for extended periods^25,75^, and in captivity they have undemanding spatial requirements, especially since they are ambush foragers with highly sedentary lifestyles that co-exist amicably in communal aggregations^32,76^. They display few of the complex animal welfare issues commonly seen in caged birds and mammals^77,78^. Reptiles also seldom transmit endotherm-centric zoonotic viruses such as bird flu, swine flu or Covid-19^79,80^.

Despite their impressive physiologies, the hands-on production of pythons differs in several important ways to mainstream livestock. For example, feeding pythons can be labour-intensive because of the current necessity to remove them from their enclosures for individual feeding (to prevent agonistic encounters with conspecifics over food). However, this labour cost may be offset against the need to only feed pythons once per week. Technical expertise and capacity is another barrier to realising the agricultural potential of pythons. The biology and husbandry requirements of pythons are poorly understood relative to many endothermic taxa. Coupled with the general fear humans have towards snakes, it may be some time before the agricultural potential of pythons is realised at the global scale.

## Conclusion

Commercial production of pythons is in its infancy, with farms receiving minimal scientific input or optimisation through formal channels for agricultural development. Even in its current relatively crude format, python farming appears to offer tangible benefits for sustainability and food systems resilience. Our study suggests that python farming can not only complement existing livestock systems, but may offer better returns in terms of production efficiencies. When compared to existing endotherm-based livestock industries, pythons are more efficient mass producers of animal protein. In countries with a cultural precedent for eating reptiles, and where food security is increasingly compromised through the impacts of global challenges such as climate change, reptiles offer an efficient, safe, and flexible source of protein. To exploit that potential, we urgently need more research into the agricultural potential of reptiles, and the most effective and humane ways to produce this novel group of livestock animals.

### Supplementary Information

Supplementary Table S1.

## Data Availability

The datasets used and/or analysed during the current study are available from the corresponding author on reasonable request.
