# Supplementary material for: Python farming as a flexible and efficient form of agricultural food security
Source: Sci Rep. 2024 Mar 14;14:5419. doi: 10.1038/s41598-024-54874-4 (PMC10940618; doi:10.1038/s41598-024-54874-4)
Supplement: Supplementary file 1 — Supplementary Table S1. [file 41598_2024_54874_MOESM1_ESM.docx]

**Supplementary material**

Table S1. Means and standard errors (SE) for growth rates in the body mass (grams/day) of Vietnamese pythons fed on different experimental diets over a 12-month period. Differences are not statistically significant. See main text for details.

| **Food type** | **Mean** | **SE** |
| --- | --- | --- |
| 100% wild rodents | 10.93 | 0.74 |
| 100% pork | 9.69 | 0.74 |
| 90% pork 10% fish pellets | 10.86 | 0.82 |
| 90% pork 10% chicken pellets | 11.94 | 1.27 |
| 80% pork 20% fish pellets | 8.53 | 0.86 |
